# Breaking Bad: Spontaneous Rupture of Incisional Hernia

**DOI:** 10.7759/cureus.56009

**Published:** 2024-03-12

**Authors:** Shiva, Faraz Ahmad, Manpreet, Konni Hemanth

**Affiliations:** 1 Surgery, King George's Medical University, Lucknow, IND

**Keywords:** incisional hernia, evisceration via incisional hernia, abdomen ventral hernia, abdominal wall surgery, spontaneous rupture of incisional hernia

## Abstract

Spontaneous bowel evisceration from a ruptured, long-standing abdominal wall hernia is a very rare complication with significant morbidity and mortality, usually occurring in incisional or recurrent groin hernias. In this case report, we elucidate an unexpected scenario of spontaneous incisional hernia rupture leading to bowel evisceration, detailing the clinical presentation, diagnostic workup, and surgical management. By highlighting this rare complication, we emphasise the significance of vigilance in monitoring hernia patients and the necessity of expedited surgical intervention to prevent complications, optimise outcomes, and minimise morbidity.

## Introduction

Incisional hernias are a common sequela of abdominal surgeries, occurring in up to 20% of patients who undergo laparotomy. While surgical repair is often indicated to prevent complications such as bowel obstruction or strangulation, spontaneous rupture leading to evisceration is a rare but potentially life-threatening complication. Despite advances in surgical techniques and perioperative care, spontaneous rupture of incisional hernias leading to evisceration remains poorly understood and infrequently reported in the literature. The underlying mechanisms contributing to this phenomenon are multifactorial, involving a complex interplay of factors such as increased intra-abdominal pressure, impaired wound healing, and intrinsic weaknesses in the abdominal wall. In this report, we present a case of spontaneous rupture of an incisional hernia leading to evisceration, highlighting the clinical presentation, diagnostic evaluation, and management strategies employed. Through this case, we aim to raise awareness of this rare but critical complication among healthcare providers and underscore the importance of early recognition and intervention in optimizing patient outcomes. Furthermore, we discuss the relevant literature surrounding the pathophysiology, risk factors, and management principles of spontaneous rupture of incisional hernias.

## Case presentation

A 74-year-old male, a chronic smoker, an alcoholic, and a daily wage labourer with no co-morbidities presented to the surgical emergency with complaints of bowel loops protruding outside the abdomen following straining while defecation. The patient does not give any history of vomiting, pain, or non-passage of faeces or flatus. He gives a history of chronic coughing and constipation. There is no history of trauma. The patient was operated on for gastric perforation 19 years ago, and a midline exploratory laparotomy with omental patching of the perforation was done. The patient developed a painless, reducible swelling that was slowly increasing in size over the anterior abdominal wall near the old operative scar two years after the surgery. He did not consult any doctor for the same, as it was symptomless.

**Figure 1:**
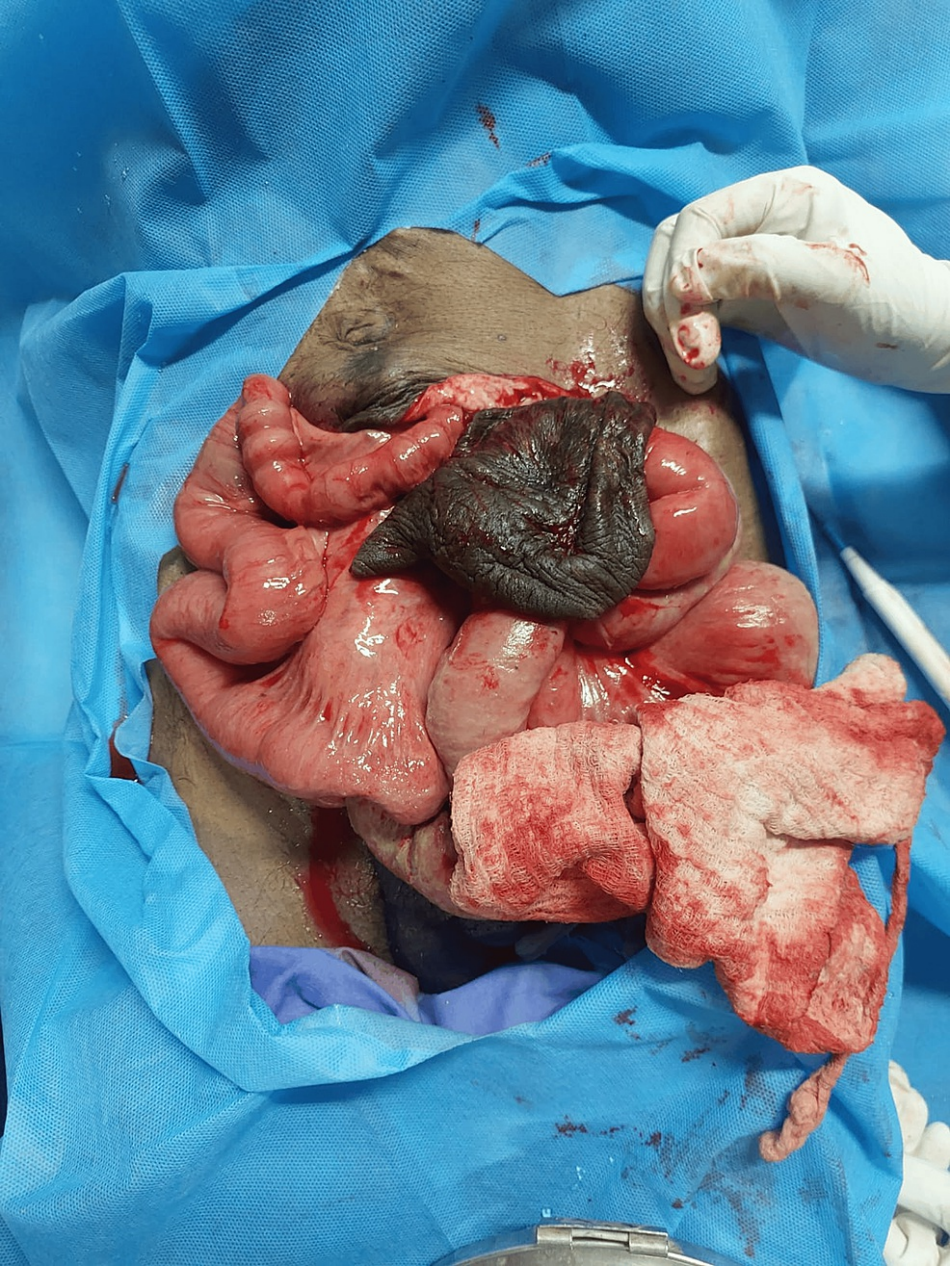
Eviscerated healthy small bowel with necrotic overlying skin and defect in the infraumbilical area

On examination, the patient was conscious, oriented, hemodynamically stable, and poorly built. The examination of other systems was within normal limits. Abdominal examination revealed a 4 cm × 5 cm incisional defect with thinned-out, necrotic overlying skin and an eviscerated, slightly oedematous, healthy small bowel approximately two metres in length with some slough in the infraumbilical region (Fig. 1).

**Figure 2:**
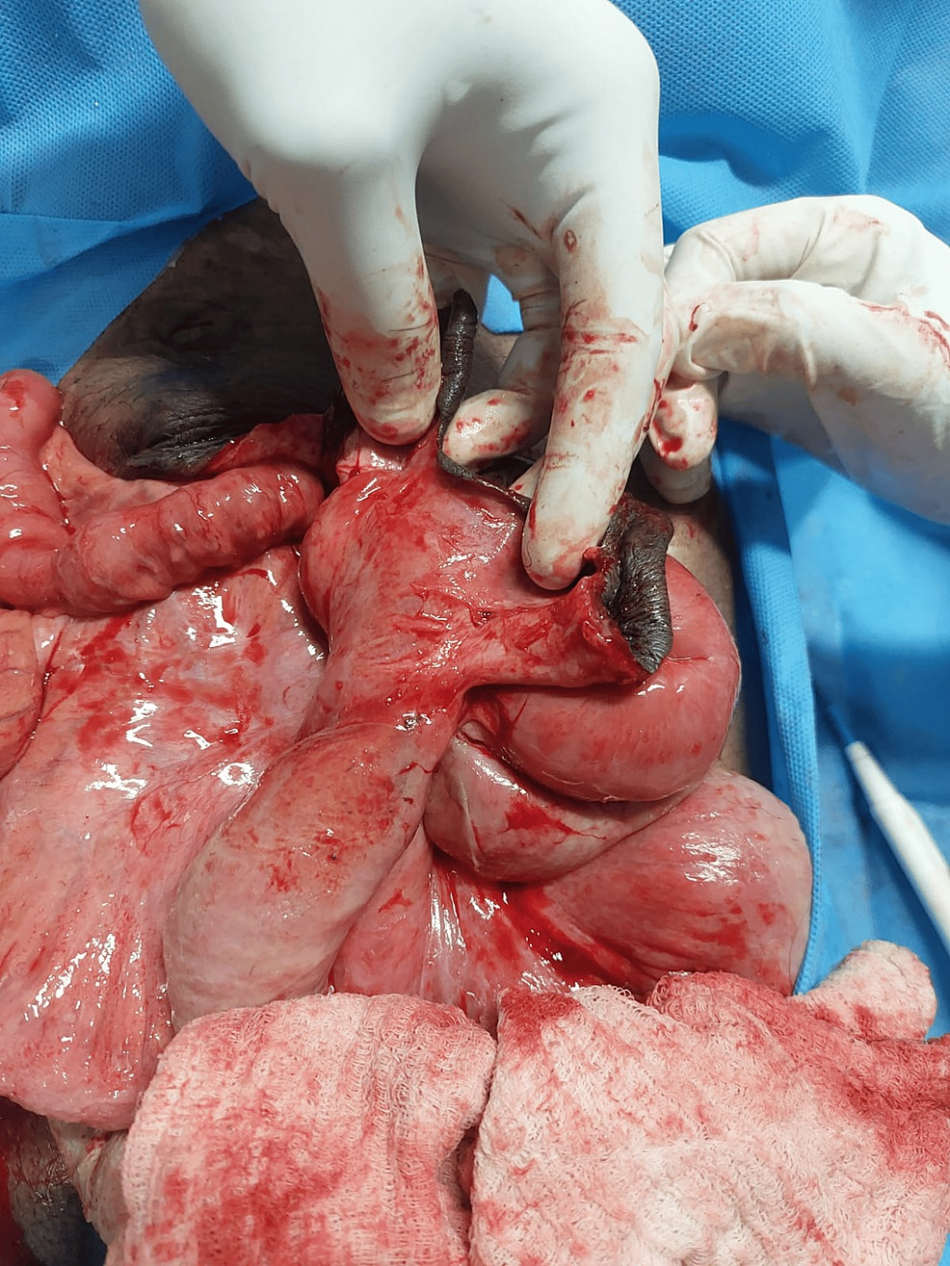
Extensive adhesions present between the prolapsed viscus and the edge of the defect in the abdominal wall

The rectal examination was suggestive of faecal loading and a posterior anal fissure. After initial resuscitation with fluids, analgesics, and antibiotics, the patient was taken up for emergency exploration. Extensive adhesions were present between the prolapsed viscus and the edge of the defect in the abdominal wall (Fig. 2).

**Figure 3:**
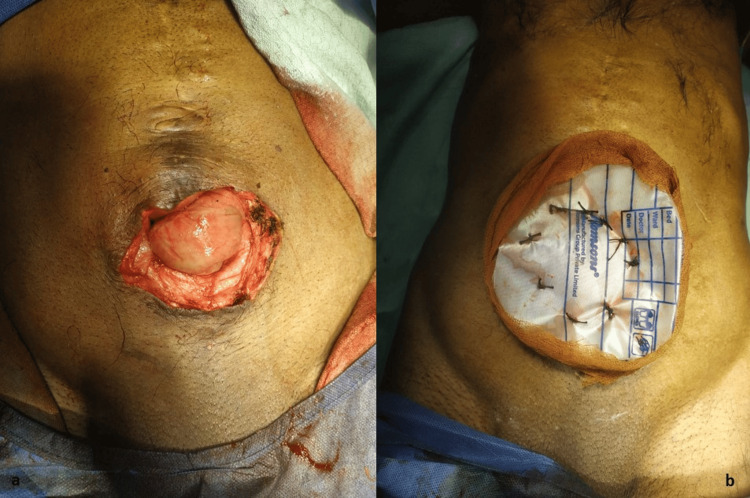
(a) Defect of size 5x6 cm visible after removing the redundant skin; (b) defect closed with urobag laparostomy

Gentle adhesiolysis was done to release the herniated loops from each other and from the hernia defect. The bowel loops were repositioned after confirming their viability and washing with normal saline. The redundant skin was excised and urobag laparostomy was done in view of excessive tension, local contamination, the possibility of abdominal compartment syndrome with primary closure, and the potential risk of infection with mesh placement (Fig. 3). 

The patient improved postoperatively without any complications and was discharged after three days with the plan of elective hernioplasty.

## Discussion

The common causes of evisceration are burst abdomens, gunshot injuries, and stab injuries. Spontaneous bowel evisceration from ventral hernia is rare and has been reported from umbilical [1, 2], incisional [3-6], parastomal [7], and inguinal hernia [8], with ascites, pregnancy [9] and obesity as predisposing factors. Spontaneous rupture of umbilical hernia and a sudden rush of fluid in patients of long-standing ascites is called "Flood Syndrome". The reported incidence of incisional hernia is 11-20% [5], with more prevalence in females attributed to lower abdominal surgeries like hysterectomies and caesarean sections (c-sections). A large hernia, contained only by its sac along with thinned-out, atrophic, avascular overlying skin, and added on by a sudden increase in intra-abdominal pressure while coughing or straining, usually leads to spontaneous rupture and evisceration.

The clinical presentation can range from just evisceration and mild congestion to severe complications like obstruction, strangulation, gangrene, perforation, or even shock. The skin defect is usually seen at the most dependent part, along with necrotic, thinned-out skin, indicating chronic neglect. The reluctance to undergo another significant surgical procedure, coupled with financial limitations causing delays in repair, results in the deterioration of the protrusion and weakening of the covering due to pressure, stretching, and ischemia, ultimately culminating in rupture.

The management necessitates prompt and comprehensive medical and surgical intervention to achieve the best possible outcomes for the patient, which includes fluid resuscitation, adequate analgesia, appropriate antibiotics for infection control, and definitive surgical treatment. Each case of spontaneous bowel evisceration warrants personalised surgical intervention, which should be based on an evaluation of risks and benefits, taking into account the patient profile, anatomical factors, the extent of the fascial defect, the degree of contamination, the proximity of the bowel, and the patient’s preferences.

The typical dilemma involves deciding between fascial re-approximation and bridging repair, mesh reinforcement or none, the position of mesh, and the use of synthetic or biological mesh. Ideally, the fascial approximation (varying from primary closure, myofascial release techniques, component separation, to free tissue flap reconstruction) is preferred over bridging repairs, and mesh reinforcement is preferred over none with placement in the underlay or retro rectus position [10]. Bridging repairs should be considered only in cases of recurrent, large, and anatomically complex defects with a guarded overall prognosis and an immediate risk of mortality.

The administration of prosthetic mesh (absorbable or permanent) repair for the condition carries the potential risk of mesh infection, whereas opting for non-prosthetic repair increases the chance of hernia recurrence due to the lack of robust natural tissues. Biosynthetic meshes combine the advantages and drawbacks of both synthetic and biological mesh types. However, the available evidence is scarce and generally does not indicate a clear superiority of biological over synthetic meshes in contaminated surgical fields, particularly concerning the risk of surgical site infections. The emergency and damage control options also include temporary abdominal closure and planned definitive fascial reapproximated closure, as was done in our case.

Potential postoperative risks include compartment syndrome, surgical site complications such as mesh infection necessitating removal, bowel erosion leading to perforation or the formation of enterocutaneous fistulas, eventration, and hernia recurrence. Early surgical intervention should be considered in patients with umbilical and incisional hernias to prevent such complications, more so in high-risk individuals like obesity, ascites, recurrent hernias, and larger defects, etc.

## Conclusions

In conclusion, this case report highlights the rare but significant occurrence of spontaneous rupture of an incisional hernia. Through careful clinical assessment and timely surgical management, the patient's condition was successfully addressed, underscoring the importance of prompt recognition and treatment of hernia complications. Continued vigilance and appropriate follow-up are essential to monitor for recurrence and ensure optimal patient outcomes in similar cases. Further studies may be warranted to explore the underlying mechanisms and risk factors associated with spontaneous rupture of incisional hernias, contributing to enhanced understanding and management of this uncommon but potentially serious condition.
